# Factors Driving COVID-19 Vaccine Hesitancy in Cameroon and Their Implications for Africa: A Comparison of Two Cross-Sectional Studies Conducted 19 Months Apart in 2020 and 2022

**DOI:** 10.3390/vaccines10091401

**Published:** 2022-08-26

**Authors:** Jerome Nyhalah Dinga, Andreas Ateke Njoh, Stanley Dobgima Gamua, Synthia Eni Muki, Vincent P. K. Titanji

**Affiliations:** 1 Michael Gahnyam Gbeugvat Foundation, Buea P.O. Box 63, Cameroon; 2 Biotechnology Unit, University of Buea, Buea P.O. Box 63, Cameroon; 3 Expanded Programme on Immunization, Ministry of Public Health, Yaounde P.O. Box 2084, Cameroon; 4 School of Global Health and Bioethics, Euclid University, Bangui BP 157, Central African Republic; 5 Faculty of Science, University of Buea, Buea P.O. Box 63, Cameroon

**Keywords:** COVID-19 vaccine hesitancy, vaccine acceptance, traditional herbal remedies, government policy, government strategy, Cameroon

## Abstract

Many efficacious COVID-19 vaccines have been approved for general use but their ability to control the disease is being undermined by slow uptake. Resources are needed to persuade people to obtain a COVID-19 vaccine. Here we compare this present study and a previous one to assess the impact of the Cameroon government’s policy and efforts to reduce COVID-19 vaccine hesitancy after one year of implementation. After obtaining ethical clearance and informed consent, 6732 participants completed a questionnaire about COVID-19 vaccine hesitancy and acceptance. It was observed that the government’s policies and efforts reduced COVID-19 vaccine hesitancy significantly, but this was not enough to ensure the herd immunity necessary to control the disease. The risk factors associated with vaccine hesitancy were the consumption of traditional herbal remedies; living in an urban setting; being female, jobless or a student; working in the education sector; being a politician/policy maker/administrator, engineer or technician; medium income; no education/primary school/secondary/high school/professional training; and working in the informal sector. In contrast, people who were male, healthcare personnel, high-income earners, participants who do not consume traditional herbal remedies, infected or knowing someone who has been infected by COVID-19, and having a chronic illness or comorbidity, were associated with COVID-19 vaccine acceptance. Participants also gave several reasons they were either hesitant or willing to take the vaccine. A more rigorous surveillance system is needed to systematically monitor drivers of vaccine hesitancy, establish tailored interventions promoting vaccine acceptance, and evaluate the impact of these interventions.

## 1. Introduction

Vaccination remains the most effective public health intervention to curb infectious disease spread and eventual eradication. Even though many COVID-19 vaccines have been approved for general use, as of 11 July 2022, the coronavirus disease remains a pandemic threatening global health and resources, having killed 253,682 people in Africa thus far. This is an indication that populations around the world are not obtaining vaccines. In the case of Cameroon, only 8% of the population has received a COVID-19 vaccine as of June 2022 [1]—a phenomenon termed vaccine hesitancy (VH). Together with technological challenges, VH has contributed to the slow uptake of vaccines [2]. Notwithstanding, through the COVAX facility, Cameroon’s central Expanded Programme for Immunization (EPI) was recently equipped with an ultra-cold chain facility [2].

VH has been defined as “a delay in acceptance or refusal of vaccines despite availability of vaccine services”, and classified as one of the top ten threats to global health in 2019 [3]. As such, it was important for countries to assess VH in their communities to determine the risk factors associated with it. This need led to a pioneer study in Cameroon in 2020 that identified COVID-19 VH as high as 85% [4]. Furthermore, misinformation regarding communication and media environment, perception of the pharmaceutical industry, and reliability and/or source of vaccine were noted as risk factors associated with VH.

For over a year now, Cameroonian authorities have implemented a policy and strategy to reduce COVID-19 VH [5]. These included official statements from the President of the Republic of Cameroon, H. E. Paul Biya, broadcast daily over national television and radio network CRTV, adverts, and talk shows on television, the radio, and social media. There were also periodic statements from renowned famous public figures and the Cameroon Academy of Sciences stating the advantages of getting vaccinated. Another strategy to encourage people to vaccinate was to ensure that only vaccinated persons were allowed access to football stadia during the just-ended Africa Cup of Nations football tournament [6]. These interventions led to more people getting vaccinated in order to watch football matches. Meetings between local authorities and community leaders were held to encourage people to take a COVID-19 vaccine. Local chiefs were also encouraged to galvanize their community members to get vaccinated [7]. The U. S. Embassy in Cameroon also released information on its website stating the availability of FDA-approved vaccines and encouraging people to get vaccinated [8]. The Ministry of Health of Cameroon also conducted two nationwide campaigns with mobile vaccination teams to educate and vaccinate those willing to take the vaccine. All these efforts are in place to reduce VH.

This study aims to assess the impact of government efforts to reduce COVID-19 VH through a national survey and comparative analysis with an earlier VH survey conducted in 2020. The secondary aims were to identify the key risk factors driving hesitancy as well as COVID-19 vaccine acceptance in the population and what role was played, if any, in terms of the consumption of traditional herbal remedies.

## 2. Materials and Methods

### 2.1. Cameroon Demographics and Health System

Information on Cameroon’s demographics and health system is found in Supplementary Material S1.

### 2.2. Study Design

This is a cross-sectional, national survey carried out from February to April 2022. It was modelled after a previously used questionnaire created by our group, and data was collected online using Google Forms and in person [4]. After obtaining consent, adults above 18 years old and residing in Cameroon were asked to complete the questionnaire. The questionnaire was composed of demographic, a close-ended and open-ended questions (Appendix A) on the region of residence, residential setting (urban/rural), age, sex, occupation, income level, education level, COVID-19 infection, consumption of traditional herbal remedies, and having a chronic disease or comorbidities, and consent to receive a COVID-19 vaccine and reasons for either vaccine hesitancy or acceptance. All questions were made compulsory to validate the response of all participants.

#### Data Collection

Participants obtained consent after explaining the purpose of the study and assuring the participants about the confidentiality and anonymity of the information requested. Adults above 18 years and residing in Cameroon were asked to complete the questionnaire online, in person, and in French or English.

The questionnaire was distributed on social media platforms and WhatsApp^®^ forums. Public figures with a large following on Facebook were contacted to voluntarily put the online questionnaire on their timeline for people to fill. The questionnaire was also distributed in national network forums of some professions to get people to fill it.

Volunteers were also recruited to assist uneducated persons in filling the form face-to-face and online. The same approach was made for persons living in rural settings around the country, using a network of friends of friends. Social distancing measures were strictly observed when administering the questionnaire face-to-face.

### 2.3. Sample Size

The Raosoft sample size calculator [9] was used to ensure the recruitment of a sufficiently large number of participants to obtain statistically significant data, given the chosen margin error (5%), confidence interval (95%), and population size (27,000,000 individuals). This gave a sample size of 385 participants. However, a larger number was surveyed to ensure proper statistical analysis across the variables. It was ensured that there were participants from each of the 10 regions of Cameroon to ascertain statistical significance in sub-groups created during the analyses of the data. The questionnaire was left open until each level of the independent variables had at least 385 participants to ensure proper statistical analysis across variables.

### 2.4. Statistical Analysis

A descriptive analysis was performed to define the distribution of demographic and other characteristics of the participants. A Chi-square test was performed to identify variable levels that would accept or reject a COVID-19 vaccine in a significant manner.

Multiple logistic regression was used to isolate risk factors associated with COVID-19 vaccine hesitancy or acceptance. Any variable level with a positive coefficient estimate value (*b*), Odds Ratio (OR) greater than 1, and a *p*-value < 0.05 was considered a significantly associated factor.

The Hosmer and Lemeshow test was used to evaluate the model’s goodness of fit.

All statistical tests were performed with Microsoft Excel 2016 and GraphPad Prism^®^ 9·3·1(471) (GraphPad Software, Inc., San Diego, CA, USA). *p*-value < 0.05 was considered significant.

### 2.5. Ethical Statement

Ethical clearance for the present study was obtained from the Institutional Review Board (IRB) of the Faculty of Health Science, University of Buea; Ref: 2020/1220-06/UB/SG/IRB/FHS.

## 3. Results

### 3.1. Socio-Demographic Characteristics

A total of 6732 participants considered to be representative of the Cameroon population filled the questionnaire. The majority of the participants were from the South West Region (*n* = 1535, 22.80%), live in an urban setting (*n* = 3787, 56.3%), of the female gender (*n* = 3751, 55.7%), above 51 years (*n* = 2486, 36.9%) and have studied at the secondary/high school/professional training level (*n* = 2868, 42.6%) (Table 1). In terms of occupation groups, most of the participants were either jobless or students (*n* = 1753, 26%) and had a low income (below 200,000 XAF/month (<$321/month)) (*n* = 4382, 65.1%) (Table 1). More than half of the participants had either been infected or know a COVID-19-infected person (*n* = 3521, 52.3%). However, only a tiny fraction had a comorbidity or chronic disease (*n* = 289, 4.3%). It was observed that a significant number of participants consumed traditional herbal remedies to control COVID-19 infection (*n* = 3909, 58.1%) (Table 1). The Chi-square test was used to identify which variable level would significantly accept or hesitate to take a COVID-19 vaccine (Table 1).

### 3.2. COVID-19 Vaccine Hesitancy Cohort Analysis

**Figure 1:**
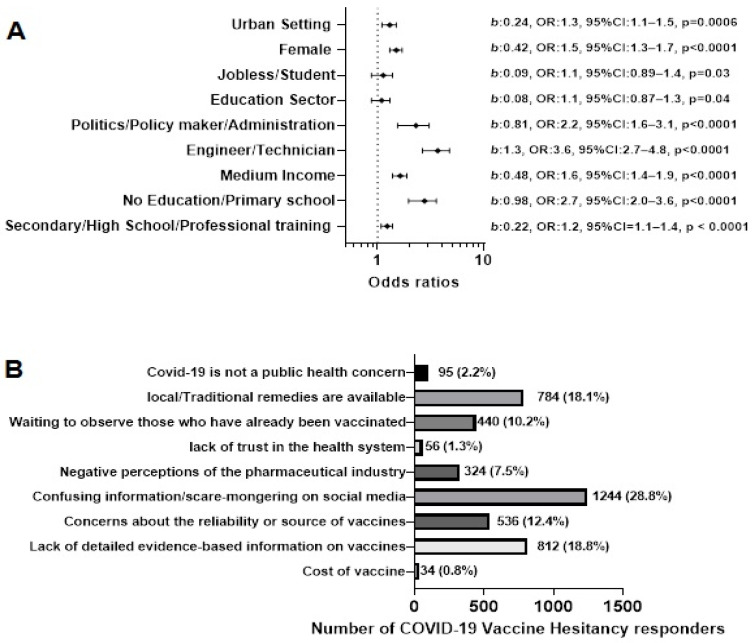
COVID-19 Vaccine Hesitancy Cohort. (**A**) Factors like to lead to COVID-19 vaccine hesitancy using multiple logistics regression. (**B**) The number of reasons for vaccine hesitancy and how many COVID-19 vaccine hesitant persons chose each reason. The different bar colours represent the different options for the participants.

The study showed that 4352 responders (64.6%) hesitated to take a COVID-19 vaccine. The Multiple Logistics Regression analysis showed that living in an urban setting (*b*: 0.24, OR: 1.3, 95% CI: 1.1–1.5, *p* = 0.0006), being a female (*b*: 0.42, OR: 1.5, 95% CI: 1.3–1.7, *p* < 0.0001), jobless or a student (*b*: 0.09, OR: 1.1, 95% CI: 0.89–1.4, *p* = 0.03), and working in the education sector (*b*: 0.08, OR: 1.1, 95% CI: 0.87–1.3, *p* = 0.04) will likely lead to COVID-19 VH. Being in politics/policy-making/administration (*b*: 0.81, OR: 2.2, 95% CI: 1.6–3.1, *p* < 0.0001) and being an engineer or technician (*b*: 1.3, OR: 3.6, 95% CI: 2.7–4.8, *p* < 0.0001) was associated with COVID-19 VH. This was also true for people with medium income (*b*: 0.48, OR: 1.6, 95% CI: 1.4–1.9, *p* < 0.0001), those that had no education or only studied to the primary school level (*b*: 0.98, OR: 2.7, 95% CI: 2.0–3.6, *p* < 0.0001), and had secondary/high school/professional training (*b*: 0.22, OR: 1.2, 95% CI = 1.1–1.4, *p* < 0.0001) (Figure 1A).

Nine reasons were presented to the VH cohort to select for hesitating to take a COVID-19 vaccine. The most frequently chosen reasons were; “Confusing information/scare-mongering on social media (*n* = 1244, 28.8%)”, “Lack of detailed evidence-based information on vaccines (*n* = 812, 18.8%)”, “Local/traditional remedies are available (*n* = 784, 18.1%)” and “Concerns about the reliability or source of vaccines (*n* = 536, 12.4%)” (Figure 1B).

### 3.3. COVID-19 Vaccine Acceptance Cohort Analysis

The percentage of participants who accepted to take a vaccine was 35.4% (*n* = 2380). Females, persons below the age of 50, healthcare personnel, and people with chronic illness or comorbidities accepted to take a COVID-19 vaccine in a significant manner (*p* < 0.0001) (Table 1).

**Figure 2:**
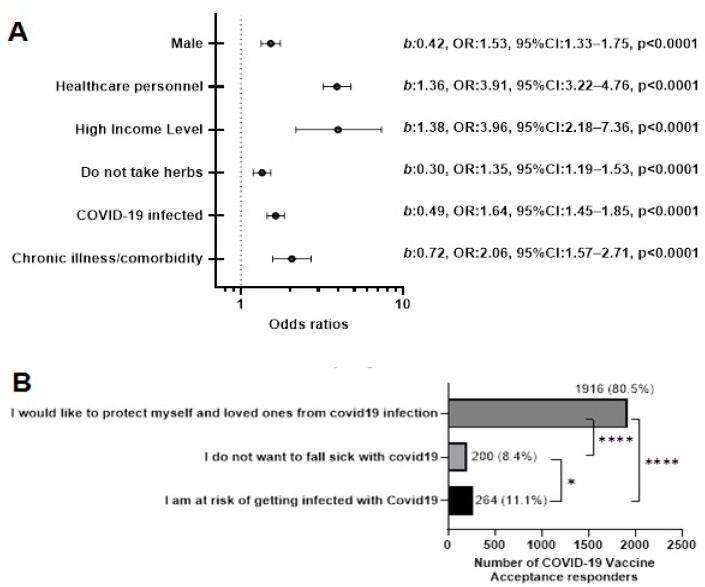
COVID-19 Vaccine Acceptance Cohort. (**A**) Using multiple logistic regression to identify factors that can likely lead to vaccine acceptance. (**B**) The reasons why persons in the vaccine acceptance cohort would give their consent to take a COVID-19 vaccine. * *p* < 0.01, **** *p* < 0.00001. The different bar colours represent the different options for the participants.

Being a male (*b*: 0.42, OR: 1.53, 95% CI: 1.33–1.75, *p* < 0.0001), healthcare personnel (*b*: 1.36, OR: 3.91, 95% CI: 3.22–4.76, *p* < 0.0001), high income earners (*b*: 1.38, OR: 3.96, 95% CI: 2.18–7.36, *p* < 0.0001), people who do not take traditional herbal remedies (*b*: 0.30, OR: 1.35, 95% CI: 1.19–1.53, *p* < 0.0001), were associated with accepting a COVID-19 vaccine (Figure 2A). The same was true for participants who have been infected or know an infected person (*b*: 0.49, OR: 1.64, 95% CI: 1.45–1.85, *p* < 0.0001), and people with a chronic illness or comorbidity (*b*: 0.72, OR: 2.06, 95% CI: 1.57–2.71, *p* < 0.0001) (Figure 2A).

Reasons given for acceptance were; (1) “I would like to protect myself and loved ones from COVID-19 infection”, (2) “I do not want to fall sick with COVID-19 infection”, and (3) “I am at risk of getting infected with COVID-19”. Out of the 2380 (35.4%) participants who obtained the COVID-19 vaccine, an overwhelming majority (*n* = 1916, 80.5%, *p* < 0.0001) said, “I would like to protect myself and loved ones from COVID-19 infection” (Figure 2B).

### 3.4. Consumption of Traditional Herbal Remedies to Control COVID-19 Infection

**Figure 3:**
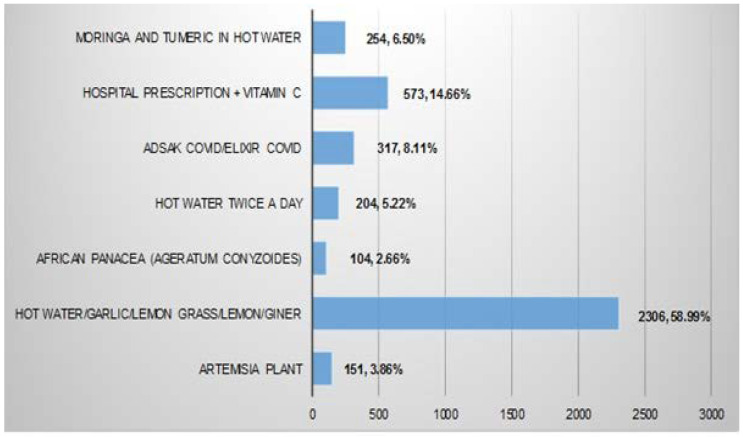
Traditional herbal remedies used to control COVID-19 infection.

This evidence-based study showed that there was a custom of taking traditional herbal remedies by people residing in Cameroon to control COVID-19 infection, as 58.1% (*n* = 3909) of the participants consumed traditional herbal remedies (Table 1). They showed COVID-19 vaccine hesitancy (*p <* 0.0001). Figure 3 shows the traditional herbal remedies and the proportion of participants who took them. The most common remedy was a hot drink mixture of lemon grass (*Cymbopogon citratus*), ginger (*Zingiber officinale*), garlic (*Allium sativum*), and lemon (*Citrus limon*) (*n* = 2306, 58.99%) and the least common being African panacea (*Ageratum* *conyzoides*) (*n* = 104, 2.66%) (Figure 3).

These are taken by people residing in Cameroon to either prevent or treat a COVID-19 infection. A total of 3909 persons in the study population said they took traditional herbal remedies for COVID-19 infection.

### 3.5. Comparative Analysis of the Present Study with an Earlier Study Carried out in 2020 [4]

The results of the present study were compared to that of an earlier study by Dinga and colleagues [4]. This was conducted to assess the impact of government policy and efforts to reduce COVID-19 VH. While 2512 people participated in the questionnaire in 2020, 6732 participants participated in the present study. Females made up 54.9% (1378) and 55.7% (3751) in 2020 and the present 2022 study. COVID-19 vaccine hesitancy stood at 84.6% in 2020 but dropped to 64.6% in 2022 (Table 2). This indicated a 25.2% reduction in VH. In 2020, according to the World Health Organization (WHO) Strategic Advisory Group of Experts (SAGE) Working Group Matrix of Determinants of vaccine hesitancy, a deductive approach was used to identify the major determinants of VH while Multiple Logistics Regression analysis was used in the present study. These risk factors were compared in Table 2.

### 3.6. Model Diagnostics and Predicted vs. Observed

**Figure 4:**
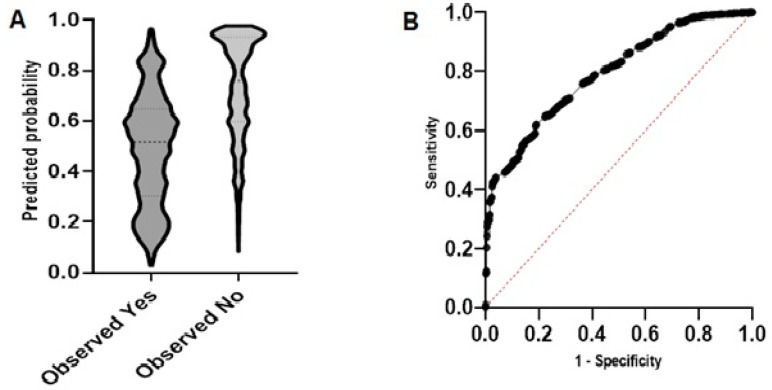
Model diagnosis. (**A**) Predicted probability graph to show how accurate the model was at predicting the outcome of the study. (**B**) The ROC curve shows the ability of the model to correctly classify each cohort of the study (area = 0.81, 95% CI: 0.97–0.81, *p* < 0.0001).

Model diagnostics was done to determine how well our data fits the selected model. The diagnostics tested the degrees of freedom and corrected Akaike’s Information Criterion (AICc) for both an Intercept-only model” and the “Selected model”. This provided us with a way to determine if the selected model did a better job fitting the data than an intercept-only model. A smaller AICc indicates a better model fit. For COVID-19 vaccine acceptance and COVID-19 vaccine hesitancy, a value of 8748 for the intercept-only model and 6905 for our select model (*p* < 0.0001) only showed that our selected model did a better job describing the observed data. Hence the variables in our selected model provided useful information about the observed data. The Predicted vs. Observed graph shows that the model performed well in classifying the group of observed COVID-19 vaccine hesitancy as the predicted probability for vaccine hesitancy was close to 1 (Figure 4A).

The Receiver Operating Characteristic (ROC) curve correctly shows the model’s ability to classify COVID-19 vaccine acceptance and COVID-19 vaccine hesitancy. The large area under the ROC curve (area = 0.81, 95% CI: 0.97–0.81, *p* < 0.0001) shows that our model had a better classification potential (Figure 4B). The *p*-value of <0.0001 indicated that the model classified the positive and negative outcomes well. The null hypothesis was rejected, and the “Selected Model is Correct” was used to check if the model fits the data well, and it did.

## 4. Discussion

It would be necessary to address VH if the implementation of effective and full coverage of a mass vaccination drive is to be achieved. Despite the successful development of numerous efficacious COVID-19 vaccines, COVID-19 continues to cause great suffering and death around the world, with African in general and Cameroon, in particular, indicating low vaccine uptake. It was earlier observed that the main contributing factor to the failure in controlling the pandemic was VH. 

An earlier study in 2020 identified VH as 84.6% among Cameroonians and its risk factors. Cameroon authorities later put a number of policies and strategies in place to tackle VH. After more than a year of implementing these policies, the present study was conducted to compare the results of an earlier study [4] to assess the impact of Cameroon government policies in reducing VH and enhancing vaccine acceptance.

Enhancing COVID-19 vaccine acceptance and tackling VH requires knowledge of evidence-based risk factors and the reasons behind a reluctance to embrace vaccination. It has been shown that the attitude toward vaccinations changes over time, necessitating the monitoring of VH and the risk factors associated with it. This will permit the mitigation of challenges that occur during vaccination programs. Even though the Cameroon government’s policy reduced VH significantly, this was not enough to ensure the 60% vaccination rate to establish herd immunity, a target endorsed by The African Union Bureau of Heads of State and Government [10]. With the advent of more contagious variants of SARS-CoV-2 such as the O family (O, O-2, O-4, O-5 and even BA.2.75), we may now require a vaccination rate above 70% to ensure herd immunity [11].

The low vaccination rate observed among those residing in Cameroon may be attributed to the consumption of traditional herbal remedies to control COVID-19. The government of Cameroon had officially authorized the marketing of four improved traditional medicines to fight against COVID-19 on the local market. These were Adsak COVID/Elixir COVID, produced by the Archbishop of Douala and Samuel Kleda, Corocur powder by Euloge Yagnigni, Palubek’s by Christine Bekono, and Soudicov Plus by Imam Modibo [12,13]. About 60% of the participants consumed traditional herbal remedies, with government-approved traditional medicines making up less than a quarter of what they consumed. This is similar to the results of a previous study that showed that the people of Cameroon have a long history of taking a variety of medicinal plants to treat flu-like symptoms [14]. Unfortunately, very little evidence-based research is being done to standardize formulation using the preliminary checklist presented here and in other studies [14]. This leaves us only with vaccines and addressing the VH risk factors as effective means of combating the COVID-19 pandemic. 

This study showed clusters of VH in urban settings, females, jobless/students, education sector, politics and administration, as well as amongst engineers/technicians. This was similar to a study in Kuwait [15]. Clusters of VH were also found amongst people with moderate, little or no education and in people with medium income, similar to that obtained in a different cross-sectional study in six Southeast Asian countries [16] and Brazil [17].

Confusing information from social media and lack of evidence-based information on vaccines were the two most popular reasons given by study participants. This outcome is similar to studies carried out in Uganda, Pakistan, Russia, and the United States [18].

Since the intention is a key driver of the uptake of health behaviours [19], the intention to receive a vaccine is likely to be greater than the actual vaccine uptake [20]. It is, therefore, necessary to identify factors associated with vaccine acceptance to support policy and communications strategies to improve vaccine uptake. A number of factors associated with vaccine acceptance were identified. The odds of accepting the vaccine for healthcare personnel were about four times that of those in the business or informal sector. The same was true for people with a high income compared to those with a low income. These results were similar to a study in Botswana and Kuwait [15,21], which showed that being male [22], at risk of getting infected [15] and having a chronic illness/comorbidity were likely to lead to COVID-19 vaccine acceptance. Compared to other studies where the intention to vaccinate was monitored over time, Pandamsee and colleagues showed that vaccine acceptance increased over time from March 2021 (*b* = 0.666; *p* < 0.001) to June 2021 (*b* = 0.709; *p* < 0.001) among Black individuals in the US [23]. This trend was also observed in another study that followed countries in the West African region and demonstrated a vaccination rate of 0.27% from February to April 2021 [10].

COVID-19 vaccines can lower the chances of infection as they work with the body’s natural defences to help develop immunity to the disease safely. This also reduces the chances of spreading disease. This knowledge may explain why an overwhelming majority of the participants chose “To protect oneself and loved ones” [24] as the reason for accepting a COVID-19 vaccine. The same trend of reasoning was observed in a different study in Sudan [25], Lebanon [26], and the United Kingdom [27].

Finally, empowering African scientists’ capacity to conduct evidence-based research on herbs and vaccines could be a bold step in the right direction to enhance confidence in the health system and vaccine safety. The examples of some African countries that are already producing their vaccines and involved in technology transfer by acquiring the mRNA technology should be copied by the rest of the continent. Increasing funds for research, empowering the network of African scientists [28,29] liaising with other agencies, community leaders and members, external partners, and donors to foster coordination and collaboration across stakeholders, could help increase confidence in the population.

Periodic surveys are also advised to monitor the impact of these strategies and identify risk factors and challenges associated with COVID-19 VH. The knowledge gained can also be translated to other diseases for implementation in the Expanded Programme on Immunization in nations across Africa and beyond.

## 5. Conclusions

The policy and strategy of the Government of Cameroon succeeded in significantly reducing VH among people residing in Cameroon. However, this reduction was not enough to establish herd immunity. A high proportion of hesitancy toward COVID-19 vaccines can significantly limit the efforts to control the disease. The conceptual framework developed by analysing VH risk factors in various sectors of life will help implement a tailored intervention to address the challenge of VH. Thus, a rigorous surveillance system must be put in place to systematically monitor THE drivers of COVID-19 vaccine hesitancy, establish tailored interventions promoting vaccine acceptance, and evaluate the impact of these interventions.

### 5.1. Strength of the Study

- The questionnaire was pretested before the commencement of the actual study.
- The study has a large sample size.
- People who have already taken a vaccine and those intending to were included in the study.
- A wide range of variables was assessed.
- Factors associated with COVID-19 vaccine hesitancy were identified by comparing two studies carried out at two different time points.
- Implementation of a vaccine hesitancy surveillance programme is feasible and beneficial.
- A thorough statistical analysis was performed on the data collected.
- Lessons could be translated to other diseases, not only in Cameroon but also in Africa and beyond.

### 5.2. Weaknesses of the Study

- Themes from the open-ended questions’ answers were manually retrieved.
- The factors being compared from the two studies were identified using two different approaches.

## Figures and Tables

**Table 1:** Socio-economic demographic characteristics of the study population. The Chi-square test was used to determine which variable level of the dichotomized variables indicated either an acceptance or hesitancy to a COVID-19 vaccine in a statistically significant manner.

Independent Variable	Level	Number (Percentage %)	*p*-Value for COVID-19 Vaccine Acceptance Using Chi-Square	*p*-Value for COVID-19 Vaccine Hesitancy Using Chi-Square
Region of Residence	**Adamaoua**	413 (6.1)	ns	<0.0001
	**Centre**	651 (9.7)		
	**East**	536 (8.0)		
	**Far North**	437 (6.5)		
	**Littoral**	431 (6.4)		
	**North**	769 (11.4)		
	**West**	424 (6.3)		
	**South**	389 (5.8)		
	**South West**	1535 (22.8)	ns	<0.0001
	**North West**	1147 (17.0)		
Residential Setting	Rural	2945 (43.7)	ns	<0.0001
	Urban	3787 (56.3)	ns	<0.0001
Gender	Male	2981 (44.3)		
	Female	3751 (55.7)	<0.0001	<0.0001
Age Group	18–30 years	2162 (32.1)	<0.0001	ns
	31–50 years	2084 (31.0)	<0.0001	ns
	51 years and above	2486 (36.9)	ns	<0.0001
Occupation	Business/Informal sector	1540 (22.9)	ns	<0.0001
	Education sector	633 (9.4)	ns	<0.0001
	Engineering/Technician	1183 (17.6)	ns	<0.0001
	Healthcare personnel	1125 (16.7)	<0.0001	
	Jobless/Student	1753 (26.0)	ns	<0.0001
	Politics/Policy-maker/Administration	498 (7.4)	ns	<0.0001
Income Level	Low income (below 200,000 XAF ($321)/month)	4382 (65.1)	ns	<0.0001
	Middle income (200,001–500,000 XAF ($322–$803)/month)	2284 (33.9)	ns	<0.0001
	High income (above 500,000 XAF (above $803)/month)	66 (1.0)	ns	ns
Education Level	No Education/Primary Education	1149 (17.1)	ns	<0.0001
	Secondary/High School level/Professional training	2868 (42.6)	ns	<0.0001
	University level	2715 (40.3)	ns	ns
Traditional herbal remedies?	No	2823 (41.9)	ns	<0.0001
	Yes	3909 (58.1)	ns	<0.0001
COVID-19 Infection?	No	3211 (47.7)	ns	<0.0001
	Yes	3521 (52.3)	ns	<0.0001
Chronic illness/Comorbidity?	No	6443 (95.7)	ns	ns
	Yes	289 (4.3)	<0.0001	<0.0001

ns = not significant. **Green** = French-speaking regions of Cameroon. **Blue** = English-speaking regions of Cameroon.

**Table 2:** Comparative analysis of two cross-sectional studies on vaccine hesitancy in Cameroon.

	Characteristics	Dinga and Colleagues [4]	Present Study
1.	No. of participants	2512	6732
	Male	45.1%	44.3%
	Female	54.9%	55.7%
2.	Vaccine Hesitancy	86.4%	64.6%
3.	Vaccine Acceptance	Not assessed	35.4%
4.	Factors Associated with Vaccine Hesitancy	Communication and media environment Perception of pharmaceutical industry, reliability Source of vaccine Cost	Living in an urban setting Being a female Jobless or a student Working in the education sector Working in politics/policy maker/administration Being an engineer or technician Having a medium income No education or studied to the primary school level Had secondary/high school/professional training.
5.	Two Major Reasons for Vaccine Hesitancy	Not assessed	Confusing information/scare-mongering on social media Lack of detailed, evidence-based information on vaccines
6.	Factors associated with Vaccine Acceptance	Not assessed	Being a male Healthcare personnel High-income earners People who do not take traditional herbal remedies Have been or know someone who has been infected by COVID-19 Chronic illness or comorbidity
7.	Two Major Reasons for Vaccine Acceptance	Not assessed	“I would like to protect myself and loved ones from COVID-19 infection.” “I do not want to fall sick with COVID-19 infection.”

## Data Availability

The data presented and analysed in this study are available with due diligence upon request from the corresponding author.

## Supplementary Materials

The following supporting information can be downloaded at: https://www.mdpi.com/article/10.3390/vaccines10091401/s1, Supplementary Material S1.

## Abbreviations

AfVANET	African Vaccinology Network
COVAX	Coronavirus diseases-19 Vaccines Global Access
COVID-19	Coronavirus diseases
EPI	Expanded Programme on Immunization
VH	Vaccine hesitancy
WHO	World Health Organization
XAF	Central African franc

## Appendix A. Study Questionnaire

Role of traditional herbal medicines in hesitancy/rejection of COVID Vaccines

Dear friend,

We researchers at the Biotechnology Unit, University of Buea and the Michael Gahnyam Gbeugvat Foundation, will like to assess ways you control or combat COVID-19 infection and your willingness to take or refuse a COVID-19 vaccine.

For almost a year now, the government of Cameroon has made COVID-19 vaccines available and put strategies in place to convince the population to take a COVID-19 vaccine. Hence, we would like to assess the perception of Cameroonians with the availability of these vaccines and what their understanding, hopes, worries and expectations of a COVID-19 vaccine are. It will also be informative to know what herbs/remedies you take for COVID-19, if any.

This survey will take about 5 min to complete. Thank you for your time and cooperation.

By filling this form, you confirm that you are 18 years of age and above and give your consent to participate voluntarily and anonymously in the study. All participants’ identifying information will be removed when analyzing data.

If you have any questions, please write to; djnyhalah@yahoo.com or vpk.titanji@yahoo.com

1 **Region of Residence:***

☐ Adamaoua

☐ Centre

☐ East

☐ Far North

☐ Littoral

☐ North

☐ North West

☐ South

☐ South West

☐ West.

2 **Residential area:***

☐ Urban

☐ Rural

3 **Gender:***

☐ Female

☐ Male

4 **Age group:***

☐ 18–30 years

☐ 31–50 years

☐ 51 and above

5 **Occupation: ***

☐ Jobless/student

☐ Medical/health care

☐ Education

☐ Engineering/Technician

☐ Business

☐ Politics/Policy maker/Administration

6 **Level of education: ***

☐ No education/Primary level

☐ Secondary/High School level/Professional training

☐ University level

7 **Income: ***

☐ Low income level

☐ Middle income level

☐ High income level

8 **Would you consent to take a covid-19 vaccine? ***

☐ Yes

☐ No

(If answer is “**Yes**” move to question 9 or if answer is “**No**” move to question 10).

9 **Reasons for accepting a COVID-19 vaccine. ***

☐ I would like to protect myself and loved ones from COVID-19 infection;

☐ I do not want to fall sick with COVID-19 infection;

☐ I am at risk of getting infected with COVID-19.

10 **Reasons for COVID-19 vaccine hesitancy:* (if applicable, choose more than one)**

☐ COVID-19 is not a public health concern;

☐ Traditional remedies are available;

☐ lack of trust in the health system/gov’t/health personnel;

☐ waiting to observe those who have already been vaccinated

☐ lack of detailed evidence-based information on vaccines;

☐ negative perceptions of the pharmaceutical industry;

☐ confusing information/scare-mongering on social media;

☐ concerns about the reliability or source of vaccines;

☐ cost of vaccine.

11 **Do you take any traditional herbal remedies against COVID-19?***

☐ Yes

☐ No

(If answer is “**Yes**” move to question 12 or if answer is “**No**” move to question 13).

12 **What traditional herbal remedies do you use against COVID-19? ***
13 **Have you ever tested positive or know anyone who has tested positive for COVID-19?** *****

☐ Yes

☐ No

14 **Do you have any chronic illness or comorbidity?** *****

☐ Yes

☐ No

*denotes compulsory question.
