# Supplementary material for: Factors Driving COVID-19 Vaccine Hesitancy in Cameroon and Their Implications for Africa: A Comparison of Two Cross-Sectional Studies Conducted 19 Months Apart in 2020 and 2022
Source: Vaccines (Basel). 2022 Aug 26;10(9):1401. doi: 10.3390/vaccines10091401 (PMC9503216; doi:10.3390/vaccines10091401)
Supplement: Supplementary file 1 [file vaccines-10-01401-s001.zip › vaccines-1858265-SI.pdf]

Supplementary Material S1

Cameroon Demographics And Health System

Cameroonians speak many languages and are a human mosaic full of over 200 ethnic groups. Its population is estimated to be above 27 million as of 2019. It has a population density of 51 per square km within a surface area of 475,650 square km. Forty-eight percent of the Cameroonian population comprises young people below the age of 15, while 3·5% are 65 and above. The country has an annual growth rate of 2.59%.

Cameroon's health map shows 189 health districts and 1800 health areas in 10 regions, including approximately 5166 private and public health facilities. Access to health services in the country was estimated to be 2·19 health facilities per 10,000 inhabitants in 2016. The health service facilities in Cameroon are organized into seven categories: general hospitals, central hospitals, regional hospitals, district hospitals, district medical centres, Integrated health centres, and ambulatory health centres.
